# The Interaction of Polyphenols and the Gut Microbiota in Neurodegenerative Diseases

**DOI:** 10.3390/nu14245373

**Published:** 2022-12-17

**Authors:** Yuan Zhang, Wanpeng Yu, Lei Zhang, Man Wang, Wenguang Chang

**Affiliations:** 1 Institute for Translational Medicine, The Affiliated Hospital of Qingdao University, Qingdao University, Deng Zhou Road 38, Qingdao 266021, China; 2 Medical College, Qingdao University, Qingdao 266021, China

**Keywords:** polyphenols, neurodegenerative diseases, intestinal microbiota, microbe–gut–brain axis

## Abstract

Polyphenols are secondary metabolites of plants and play a potential role in the prevention and treatment of neurodegenerative diseases (NND) such as Alzheimer’s disease (AD) and Parkinson’s disease (PD) due to their unique physiological functions such as acting as antioxidants, being anti-inflammatory, being neuroprotective, and promoting intestinal health. Since dietary polyphenols exist in plant foods in the form of glycosylation or esterification or are combined with polymers, they need to undergo extensive metabolism through phase I and phase II biotransformations by various intestinal enzymes, as well as metabolism by the intestinal microbiota before they can be fully absorbed. Polyphenols improve intestinal microbiota disorders by influencing the structure and function of intestinal microbiota, inducing beneficial bacteria to produce a variety of metabolites such as short-chain fatty acids (SCFAs), promoting the secretion of hormones and neurotransmitters, and playing an important role in the prevention and treatment of NND by affecting the microbe–gut–brain axis. We review the ways in which some polyphenols can change the composition of the intestinal microbiota and their metabolites in AD or PD animal models to exert the role of slowing down the progression of NND, aiming to provide evidence for the role of polyphenols in slowing the progression of NND via the microbiota–gut–brain (MGB) axis.

## 1. Introduction

Polyphenols, as secondary metabolites of plants, are widely found in fruits, vegetables and plant-derived foods. Polyphenols are composed of at least two benzene rings and one or more hydroxyl substituents, mainly including phenolic acids; flavonoids; (1,2-thendiyl)-1,1-bisbenzene; coumarin; tannins; etc. [1]. Due to their unique physiological functions, such as antioxidant, anti-inflammatory, neuroprotective and promoting intestinal health, polyphenols exert a potential role in the prevention and treatment of neurodegenerative diseases (NND), such as Alzheimer’s disease (AD) and Parkinson’s disease (PD) [2,3]. Polyphenols have been demonstrated to modulate brain function mainly via affecting cerebral blood flow; impaired neurogenesis and synaptic plasticity; aberrant protein aggregation; and regulating neuro-inflammation, mitochondrial dysfunction, and oxidative stress [4,5,6].

Although polyphenols are highly abundant in the diet, they are poorly bioavailable in the body. Polyphenols can be divided into bound and free phenols. Free phenols refer to phenolic compounds that do not interact with other macromolecules physically or chemically. They can be hydrolyzed by small-intestinal esterase and then absorbed to affect human health. However, free phenols only account for 5–10% of total phenols, while 90–95% are bound phenols, which need to be decomposed into small molecule metabolites through the action of intestinal microorganisms so that they may enter the liver and intestine blood circulation [7]. Since dietary polyphenols exist in plant foods in the form of glycosylation, esterification or combined with polymers, they need to undergo extensive metabolism through phase I and phase II biotransformations by various intestinal enzymes to change their original structure after absorption, and then the small part of secondary bioactive metabolites are bioavailable, entering systemic circulation [8,9]. However, most of the metabolites can only be fully absorbed after biotransformation of intestinal microbiota to improve their biological activity [10]. Many microorganisms colonize the colon of the body, such as *Firmicutes*, *Bacteroidetes*, *Actinobacteria*, *Proteobacteria*, and *Verrucomicrobia*. *Firmicutes* and *Bacteroidetes* account for more than 90% of bacterial species in normal healthy humans, but the composition of the gut microbiome is highly influenced by diet, disease and drugs. Intestinal microecological dysregulation is observed in patients with NND; for example, the richness and diversity of intestinal microbiota are reduced, the relative abundance of beneficial bacteria is decreased, and the abundance of inflammation-related taxa is increased [11]. In addition, polyphenols induce beneficial bacteria to produce a variety of metabolites, including short-chain fatty acids (SCFAs), and to promote the secretion of hormones and neurotransmitters (such as serotonin and gamma-aminobutyric acid), which play an important role in the prevention and treatment of neurodegenerative diseases by affecting the microbiome–gut–brain axis [12]. Therefore, the effects of polyphenols on human health mostly depend on the intervention of intestinal microorganisms [13,14]. The changes in the composition of these bacteria and their metabolites have a very important impact on the pathology of neurodegenerative diseases, such as anti-neuroinflammation and antioxidative stress. Polyphenol compounds and their metabolites can slow the progression of neurodegenerative diseases by improving the disorder of gut microbiota. Here, we review the mechanism of polyphenols’ metabolism in the gut and the effects by which polyphenols improve brain function by regulating intestinal flora and its metabolites, aiming to provide evidence for the role of polyphenols in slowing the progression of NND via the microbiota–gut–brain (MGB) axis.

## 2. Metabolism of Polyphenols in the Gastrointestinal Tract

After dietary polyphenols are ingested, some of them are initially absorbed to a low degree in the stomach. Only a small part of the polyphenols is absorbed in the small intestine (mainly free polyphenols). Dietary polyphenols are poorly absorbed in the body and are generally metabolized extensively in enterocytes and the liver through phase I and II biotransformations. Polyphenols are present in foods commonly conjugated with sugars or organic acids or are present as unconjugated oligomers. Approximately 5% to 10% of polyphenols with monomer and dimer structures may be absorbed directly by the small intestine. Some phenolic polymers can be hydrolyzed by α-rhamnosidase, β-glucosidase and β-glucuronidase to complete the phase I biotransformation [15,16]. The released aglycogens can be transferred to enterocytes by passive diffusion or reach the liver through the portal vein circulation, where they undergo phase II biotransformation in enterocytes and hepatocytes. In there, polyphenolic aglycones are combined with glucuronates, sulfates and/or methyl moieties and are converted to O-glucuronate or O-sulfonate forms by methylation, sulfation, hydroxylation and glucosidation, which are then distributed to organs and excreted in the urine. Because of their structural characteristics, most dietary polyphenolic compounds can be directly metabolized in phase II biotransformation instead of phase I biotransformation [8,9]. However, the rest of the unabsorbed polyphenols (mainly bound polyphenols) reach the colon, where they are decomposed, released and absorbed and are subjected to intestinal microbiota function. Polyphenols promote the growth of beneficial microbes in the gut microbiome, such as *Lactobacillus* and *Bifidobacterium*, which are two major beneficial probiotics that benefit human health and indirectly reduce the number of pathogenic microbial species, such as *Clostridium histolyticum* and *Clostridium perfringens* in *Clostridium* [17,18]. In addition, intestinal microbiota can metabolize polyphenols with high molecular weight into more bioactive metabolites to improve their bioavailability. Studies have shown that 90% of ingested polyphenols are converted into bioavailable products under the action of gut microbiota (Table 1).

For example, curcumin is a hydrophobic polyphenol that is extracted from the rhizome of Curcuma longa. Curcumin undergoes extensive phase I and phase II biotransformation in intestinal cells and the liver. When ingested, curcumin is reduced to dihydrocurcumin (DHC), tetrahydrocurcumin (THC), hexahydrocurcumin (HHC) and octahydrocurcumin (OHC) in enterocytes and hepatocytes by alcohol dehydrogenases [19]. The metabolites are further converted to curcumin glucuronide by uridine 5′-diphospho-glucuronyltransferases (UGT) or converted into curcumin sulfate by sulfotransferases (SULT). Furthermore, the transformation of curcumin in the gut requires several enzymes produced by the gut microbiota. For example, the firmicute *Blautia* sp. MRG-PMF1 metabolizes curcumin to produce demethylcurcumin and bisdemethylcurcumin through a demethylation reaction. *Escherichia fergusonii* (ATCC 35469) and two *E. coli strains* (ATCC 8739 and DH10B) metabolize curcumin to produce dihydrocurcumin, tetrahydrocurcumin and ferulic acid [20]. In addition, studies have shown that more intestinal microflora have been found to produce a variety of metabolites through demethylation, reduction, hydroxylation and acetylation. These metabolites have been shown to have therapeutic effects on NND through their antioxidant and anti-inflammatory effects [21].

Quercetin and its derivatives, such as rutin, are common dietary flavonoids. Quercetin and rutin are absorbed at the level of the small intestine through phase I and phase II biotransformation. Quercetin is mostly present as glycosides, such as rutin (quercetin-3-O-rutinoside, rutoside). Prior to being absorbed by the intestine, rutin must primarily be deglycosylated into quercetin aglycon by the lactase phlorizin hydrolase, a β-glucosidase residing on the luminal side of the brush border in the small intestine (phase I biotransformation) [22]. Quercetin aglycon undergoes phase II biotransformation in enterocytes and generates glucuronated, sulfated and methylated metabolites through the function of UGTs, SULTs and catechol-O-methyltransferases (COMTs). Quercetin and rutin, which are not absorbed by the intestine, are further metabolized by the microflora in the colon. Rutin is initially hydrolyzed to quercetin by β-glucosidase derived from intestinal microbiota, and then quercetin is produced by *Eubacterium ramulus*, *Clostridium orbiscindens*, *Eubacterium oxidoreducens* and *Butyrovibrio* spp. through C-ring fissions and dehydroxylation to produce low molecular weight phenolic compounds that can easily be absorbed. In addition, several bacterial strains, including *Bacteroides fragilis*, *Eubacterium ramulus*, *Clostridium perfringens*, *Bacteroides JY-6*, *Bifidobacterium B-9*, *Lactobacillus L-2* and *Streptococcus S-2*, are responsible for transforming quercetin into homoprocatechuic, protocatechuic, 4-hydroxybenzoic and 3-(3-hydroxyphenyl) propionic acids [23,24,25].

Daidzein and genistein are known as common isoflavones with estrogen-like effects. The hydrolysis of β-glucosidase is the initial and very important step in the metabolism of isoflavones to produce bioactive substances in vivo. Hydrolysis is a process that occurs throughout the intestinal tract, resulting in the production of the respective aglycones from their glycosylated forms due to the function of β-glucosidases in the intestinal tract [26]. After ingestion, daidzein and genistein are initially hydrolyzed by β-glucosidase secreted by intestinal microbiota colonizing the human ileum and colon, including *Lactobacillum*, *Bifidobacterium* and *Bacteroides*. Deglucosylated daidzein and genistein are subdivided by *Lactococcus strains, E. faecium* INIA P455 and *L. paracasei* INIA P461. These strains produce dihydrodaidzein (DHD) and dihydrogenistein (DHG) through hydrogenation reactions. Furthermore, *Eggerthella* sp. YY7918 can convert daidzein and DHD into S-equol. Recently, studies have shown that *Eubacterium ramulus* isolated from human feces is able to undergo anaerobic C-ring cleavage to convert genistine into 6′-hydroxy-O-desmethylangolensin, followed by 2-(4-hydroxyphenyl)-propionic acid, and directly convert daidzein into O-desmethylangolensin (O-DMA). Other bacteria, such as *Eubacterium ramulus* and *Clostridium* sp. HGH 136, can convert daidzein to O-DMA [8,27,28].

Resveratrol is a polyphenolic compound mainly derived from peanut, grape (red wine), knotweed, mulberry and other plants. Resveratrol is chemically known as (E)-3,5,4-trihydroxystilbene. It is a nonflavonoid polyphenolic compound. Most resveratrol undergoes phase II biotransformation in enterocytes and the liver, including glucuronidation, sulfation and metabolism by gut microbes. At the intestinal level, the conjugation of resveratrol with glucuronic acid is catalyzed by UGT to produce resveratrol-3-O-glucuronide (R3G) and resveratrol-4′-O-glucuronide (R4G). Resveratrol is metabolized by SULT to produce resveratrol-3-O-sulfate (R3S), resveratrol-4′-O-sulfate (R4S) and resveratrol disulfates (RdS) [29]. Intestinal bacteria contribute to metabolizing resveratrol. Initially, *Bifidobacteria infantis* and *Lactobacillus acidophilus* can hydrolyze the glucoside moieties of plant glycosydes from the resveratrol precursor piceid to produce resveratrol. Resveratrol is then hydrogenated by *Slackia equolifaciens* and *Adlercreutzia equolifaciens* to convert into dihydroresveratrol (DHR), which is partially absorbed and further metabolized into monosulfate DHR or monoglucuronide DHR that can easily be eliminated in urine [30].

Anthocyanins, a kind of flavonoid compound, are water-soluble pigments widely existing in plants. Anthocyanins are usually bound to one or more glucose, rhamnose, or galactose through glycosidic bonds. Anthocyanins are mainly absorbed in the stomach and small intestine. Studies have shown that anthocyanin glycosides can be absorbed directly into the small intestine [31,32]. However, the unabsorbed anthocyanins can be decomposed into phenolic acids and monosaccharides under the action of colon microorganisms such as *Lactobacilli* and *Bifidobacteria*. Studies have shown that anthocyanins promote an increase in beneficial bacteria, including *Lactobacilli* and *Bifidobacteria*, while reducing pathogenic bacteria, including *Staphylococcus aureus* and *Salmonella typhimurium*. In addition, cyanidin-3-O-glucoside produced by phase I and phase II biotransformation of anthocyanins can be further deglycosylated by gut bacteria, including *Eubacterium ramulus* and *Clostridium saccbarogumia,* to produce protocatechuic acid (PCA) and gallic, syringic, vanillic and p-coumaric acids. These phenolic acids are produced to exert their antioxidant activity directly in the gut [33].

More complex polyphenols, especially oligomeric polyphenols and polymeric structures such as concentrated or hydrolyzed tannins, are almost unchanged when they reach the colon of the organism but can be further metabolized by the gut microbiota. Here, they undergo microbial enzyme transformations, including C-ring cleavage, decarboxylation, dehydroxylation and demethylation, to produce relatively simple compounds such as phenolic acids and hydroxycinnamate esters.

For example, ellagitannins are hydrolyzable tannins that are widespread in numerous plant species and play a very important role in human nutrition, such as antioxidant and anti-inflammatory functions. The ellagitannins hydrolyze to release ellagic acid (EA) and then reach the gut, where they undergo microbial metabolism, lose one of the lactone rings, and are gradually dehydrolyzed to urolithins (Uros), which are absorbed by the body [34]. In addition, Uros can also be combined with glucuronic acid and sulfuric acid to generate glucuronide and sulfate derivatives. Natural Uros are not common in nature [35]. They are often distributed in the blood, urine, bile, feces and colon of humans or other lactating animals in the form of sulfation, glycosylation and methylation. However, the absorption of ellagitannins and EA in the gastrointestinal tract is poor, and their bioavailability is low. Unabsorbed EA is metabolized into absorbable Uros by the gastrointestinal microbiota of mammals. Recent studies have shown that the *Gordonibacter* genus and *Clostridium coccoides* group are responsible for the generation of Uros by the metabolism of EA [36].

Proanthocyanidins belong to a group of plant polyphenols. Proanthocyanidins consist of condensed flavan-3-ol units with high structural complexity. Proanthocyanidins are structurally formed by the combination of different amounts of catechin or epicatechin. The common proanthocyanidins are catechins, epicatechins or the dimers synthesized from catechin and epicatechin. In addition, there are trimers, tetramers and so on until the decamer. According to the size of polymerization, the dipentamer is usually called oligomeric proanthocyanidins (OPCs), and the pentamer is called high polyproanthocyanidins (PPCs). Among all polymeric PAs, OPCs have the strongest functional activity. The monomers and oligomers of proanthocyanidins enter enterocytes, and hepatocytes are glucosidated by UGTs in the endoplasmic reticulum. Sulfation and methylation mediated by cytosolic SULTs and COMTs can also occur in enterocytes and hepatocytes. Epigallocatechin gallate (EGCG) and epigallocatechin (EGC) are metabolized to glucuronides, especially by the human liver and intestinal UGT isoforms [37,38,39]. Proanthocyanidin catabolism begins with interflavan bond cleavage or C-ring cleavage, followed by A-ring oxidation, dehydroxylation and beta-oxidation. EGCG could be catalyzed into EGC and gallic acid (GA) through hydrolysis at C3, and a similar reaction could take place for epicatechin gallate (ECG), which could be catalyzed into epicatechin (EC) and GA. Proanthocyanidins are biotransformed by *Adlercreutzia equolifaciens JCM 14793T*, *Eubacterium* sp. *SDG-2*, *Eggerthella lenta rK3* and *Eggerthella lenta CAT-1* and from the phylum actinomycetes, which belong to the phylum *Actinobacteria*, through the cleavage of the C-ring and dihydroxylation reactions to produce (−)EGC, (−)-gallocatechin (GC), (±)EC and (±)-catechin (C) [39].

## 3. Effects of Polyphenols on Neurodegenerative Diseases by Gut Microbiota Metabolism

**Figure 1:**
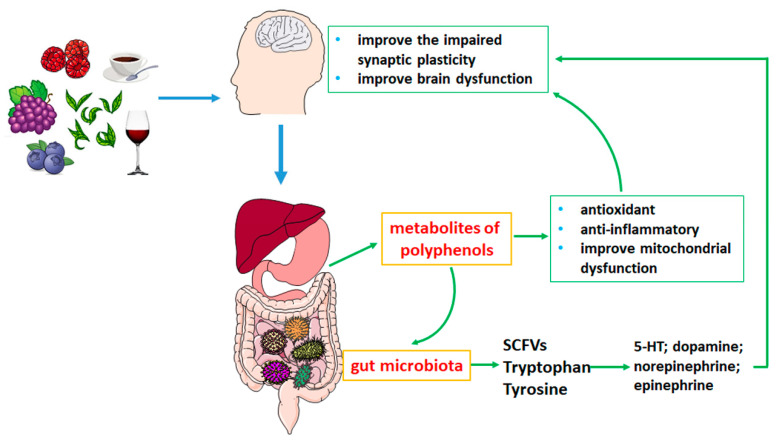
Effects of polyphenols on neurodegenerative diseases by gut microbiota metabolism.

Intestinal microecology is dysfunctional in NND patients; for example, the richness and diversity of intestinal microbiota are reduced and the relative abundance of beneficial bacteria is decreased, resulting in the potential reduction of synthesis of SCFAs (acetic acid, propionic acid, butyric acid) and an increase in the abundance of inflammation-related taxa. The changes in the composition of these bacteria and their metabolites have a very important impact on the pathology of neurodegenerative diseases, such as anti-neuroinflammation and antioxidative stress. Polyphenol compounds and their metabolites can slow the progression of neurodegenerative diseases by improving the disorder of gut microbiota (Figure 1 and Table 2).

### 3.1. Polyphenols Affect the Composition of Gut Microbiota

#### 3.1.1. Isoorientin

Isoorientin (3′,4′,5,7-tetrahydroxy-6-C-glucopyranosyl flavone) is a natural flavonoid widely found in Passiflora edulis, corn silk, bamboo and gentian leaves that has strong antioxidant and anti-inflammatory properties. Studies have demonstrated that isoorientin reduces oligomeric Aβ in the hippocampus and cortex and alleviates synaptic dysfunction and spatial memory deficits in APP/PS1 mice; therefore, it has potential therapeutic efficacy in AD [40,41]. More evidence has demonstrated that isoorientin treats neurodegenerative diseases by modulating the gut microbiota. Zhang et al. demonstrated that isoorientin decreased Aβ plaque deposition in the cortex and hippocampus of AD mice and significantly decreased TNF-α, IL-6, iNOS and COX-2 and increased IL-4 and IL-10 in AD mice [42]. At the same time, isoorientin treatment promoted the distribution of the class *Mollicutes*, family *Prevotellaceae*, and genus *Prevotellaceae* UCG 001 in the fecal microbiota and the phylum *Proteobacteria* (*Pasteurellales*: *Pasteurellaceae*) in the cecal microbiota of AD mice [42]. The results showed that isoorientin serves as a nutraceutical agent and a lead drug candidate for neurodegenerative diseases.

#### 3.1.2. Quercetin/Quercetin-3-O-Glucuronide

Evidence shows that quercetin exerts neuroprotective effects on neurodegenerative disorders due to its antioxidant and anti-inflammatory properties. Although quercetin showed comparatively low oral bioavailability, it can have a neuroprotective function through its interaction with gut microbiota. Xie et al. reported that quercetin reduced myelin and axonal damage in a diabetic peripheral neuropathy (DPN) rat model; earlier, they used streptozocin to induce an AD model, reversed the ROS production level, and increased the diversity of gut microbiota among groups [43]. Quercetin may function by decreasing the abundance of potential ‘pathogenic’ bacteria, such as f_*Porphyromonadaceae*, f_*Oxalobacteraceae*, g_*Oxalobacter* and g_*Klebsiella*, which were positively correlated with DPN phenotypes and ROS production levels, while enriching the potential ‘probiotic’ bacterial taxa, such as p_*Actinobacteria* and c_*Actinobacteria*, which were negatively correlated with DPN phenotypes and ROS production levels, to regulate intestinal microbiota and play a neuroprotective role in DPN rats. Therefore, quercetin therapy plays a role in the neuroprotective mechanism of AD by reversing DPN-induced intestinal disorders through enrichment of *Actinobacteria* at the phylum and class levels.

Quercetin-3-O-glucuronide (Q3G) is the primary metabolite of quercetin in vivo.

Studies have shown that quercetin-3-O-glucuronide plays a neuroprotective role in AD by reducing Aβ accumulation and tau phosphorylation and attenuates cognitive dysfunction in Aβ42-induced AD-like mice [44]. Moreover, quercetin-3-O-glucuronide supplementation could restore intestinal microbiota dysbiosis. The abundance of *g_Alistipes* and *g_Rikenella* was markedly increased, and *g_Barnesiella* and *g_Lactobacillus* were significantly reduced in the Aβ group, which correlated with the levels of inflammatory factors in the brain. However, quercetin-3-O-glucuronide treatment restored the abundance of these gut microbiota to normal levels, which was related to preventing neuroinflammation. In addition, quercetin-3-O-glucuronide restores the Aβ42-induced reduction in SCFAs, which is related to changes in the gut microbiota [44].

#### 3.1.3. Fisetin

Fisetin is a bioactive flavonoid abundant in various vegetables and fruits. Fisetin has been shown to be a potential drug for the treatment of NND, such as AD, PD and amyotrophic lateral sclerosis (ALS), due to its antioxidant, anti-inflammatory and neuroprotective effects and has been validated in a series of preclinical models [45]. In recent years, fisetin has been reported to prevent neurodegenerative disorders by regulating the diversity and distribution of intestinal microbiota. For example, fisetin changed the number, diversity and distribution of gut microbiota in a 1-methyl-4-phenyl-1,2,3,6-tetrahydropyridine (MPTP)-induced mouse model of PD [46]. Chen et al. reported that fisetin treatment improved MPTP-induced behavioral impairments in mice and attenuated dopaminergic neurodegeneration in the substantia nigra (SN) and striatum of PD mice. Moreover, fisetin treatment changed the distribution and diversity of gut microbiota in a PD mouse model. Fisetin treatment significantly increased the abundance of *Lachnospiraceae* and significantly reduced the abundance of uncultured_bacterium_g_Escherichia-Shigella and uncultured_bacterium_g_Bacillus. Previous studies have shown that the increased abundance of *Lachnospiraceae* may produce enhanced SCFA levels and exert neuroprotective effects. Therefore, studies have indicated that fisetin achieves the purpose of treating PD by increasing the abundance of *Lachnospiracea*, which might be related to the increased level of SCFAs.

#### 3.1.4. Anthocyanins

Anthocyanins are a class of flavonoids found in red and purple berries that exhibit anti-neuroinflammatory and antioxidative stress properties in central nervous system (CNS) disorders. Recent evidence indicates that the prevention of neurodegenerative diseases attributed to anthocyanins may be associated with modulation of gut microbiota. Marques et al. demonstrated that blackberry anthocyanin-rich extract (BE) could attenuate the neurologic complications of obesity, which was associated with modulating gut microbiota composition [47]. Anthocyanin treatment reduced TCK-1 expression in the hippocampus of high-fat diet-induced rats and inhibited the passage of LPS into the circulation, whereas it increased *Pseudoflavonifractor* and *Sporobacter*. Furthermore, anthocyanin increased the levels of tryptophan and kynurenic acid in the urine of high-fat diet-induced rats. The kynurenic acid generated from tryptophan metabolism can be used as an antagonist at excitatory amino acid receptors and has been reported to be associated with psychiatric diseases [48].

This result indicates that anthocyanins counteract diet-induced neuroinflammation by altering the composition of the gut microbiota to stimulate the kynurenine pathway of tryptophan metabolism. Another study showed that anthocyanins attenuated oxidative stress induced by a high-fat diet in mice by increasing SOD and GSH-Px activity and increased the levels of 5-hydroxytryptamine (5-HT) and dopamine in the hippocampus of mice while also increasing the diversity of specific bacteria and their metabolites [49]. A study showed that anthocyanin treatment enhanced the abundance of *Bifidobacterium*, *Lactobacillus*, *Roseburia*, *Faecalibaculum*, *Parabacteroides and Ruminiclostridium* and reduced the abundance of *Staphylococcus*. Moreover, previous studies have shown that *Roseburia*, *Faecalibaculum* and *Parabacteroides* are related to the production of SCFAs, *Staphylococcus* is related to gut inflammation, and *Ruminiclostridium* is related to gut–brain axis function. Therefore, anthocyanidin-rich dietary interventions are a promising defense against oxidative damage, neuroinflammation and neurodegeneration in humans.

#### 3.1.5. Curcumin

Curcumin is the most studied polyphenolic compound for the treatment of neurodegenerative diseases. Previous studies have shown that the application of curcumin in AD and PD therapy may be its antiaggregative effect on pathological proteins [50]. Growing evidence suggests that curcumin treatment can increase the diversity of gut microbiota and modulate the abundance of several key bacterial species associated with NND development. For example, curcumin treatment significantly decreased the relative abundances of *Bacteroidaceae*, *Prevotellaceae* and *Lactobacillaceae* and increased the relative abundance of *Rikenellaceae* at the family level. Furthermore, curcumin administration reduced the relative abundances of *Prevotella*, *Bacteroides* and *Escherichia/Shigella* and increased the relative abundance of *Parabacteroides* at the genus level [51,52]. Therefore, these microflorae are reported to be associated with the development of AD [52,53,54]. Moreover, curcumin is biotransformed to a variety of metabolites by gut microbiota, which may contribute to the therapeutic effect of AD. For instance, curcumin is biotransformed by the gut microbiota of AD mice through reduction, demethoxylation, demethylation and hydroxylation to produce eight kinds of metabolites, demethylcurcumin, demethoxylated curcumin, hydroxylated curcumin, dihydrocurcumin, hexahydrocurcumin, demethylated hexahydrocurcumin, bisdemethylated hexahydrocurcumin and dehydroxylated hexahydrocurcumin, which have neuroprotective functions [51]. Previous studies have shown that demethylcurcumin and bisdemethoxycurcumin produced by human intestinal microbiota can increase the activity of the Aβ-degrading enzyme neprilysin, while parent curcumin cannot. Ahmed et al. reported that bisdemethoxycurcumin and demethoxycurcumin showed stronger memory-enhancing activity than parent curcumin in Aβ-induced AD rat models. In addition, a study showed that Di-O-demethylcurcumin exerts a neuroprotective role by preventing Aβ (25-35)-induced mitochondrial and endoplasmic reticulum-mediated apoptosis in neuroblastoma cells.

In addition, curcumin has been reported to improve motor deficits and glial cell activation in PD patients by inhibiting α-synuclein aggregation (α-syn). However, these functions were also closely related to the regulation of intestinal microbiota disorder by curcumin. Curcumin treatment increased the level of levodopa (dopa) in the brain of the MPTP-induced PD mouse model, as well as elevated the levels of *Muribaculaceae*, *Lactobacillaceae*, *Lachnospiraceae* and *Eggerthellaceae* but reduced the levels of *Aerococcaceae* and *Staphylococcaceae* [55]. Cui et al. also reported that curcumin significantly increased the levels of tyrosine, methionine, sarcosine and creatine in the serum of PD mice [55]. In particular, tyrosine plays an important neuroprotective role in PD pathology. The changes in these metabolites were closely related to the changes in the abundance of intestinal microbiota modulated by curcumin. For instance, tyrosine identified strong correlations with the levels of *Lactobacillaceae*, *Aerococcaceae* and *Staphylococcaceae*, and sarcosine and creatine were negatively correlated with the levels of *Staphylococcaceae*.

#### 3.1.6. Resveratrol

The direct evidence for the effect of resveratrol on NND by affecting gut microbiota has not been widely reported. However, previous evidence shows that resveratrol has a significant neuroprotective role in vivo and in vitro due to the effects of anti-oxidative stress and anti-inflammation. Resveratrol promotes non-amyloidogenic processing of the amyloid precursor protein (APP) to reduce the level of Aβ in the brain of AD animals, and it reduces microglial and astrocytic activation by inhibiting the activation of c-Jun amino-terminal kinase (JNK) and glycogen synthase kinase 3β (GSK-3β), thus alleviating cognitive dysfunction in animal models of AD [56,57]. In addition, studies have shown that resveratrol plays a neuroprotective role in PD rats by activating antioxidant pathways and inhibiting dopaminergic neuron damage [58,59]. There is increasing evidence that resveratrol has beneficial functions in CNS diseases through the gut–brain axis. Resveratrol affects the level of glucagon-like peptide 1 (GLP-1) and 5-hydroxytryptamine (5-HT) in the intestine by regulating the composition of intestinal microbiota such *Lactobacillus* and *Bifidobacterium* to prevent CNS inflammation [60,61]. Li et al. report that resveratrol functional selenium nanoparticles (Res@SeNPs) can significantly improve cognitive dysfunction of AD through restoring gut microbiota disorder [62]. Studies have shown that resveratrol surface-functionalized selenium can increase the ability of inhibiting Aβ aggregation and ROS formation better than resveratrol alone. Furthermore, Res@SeNPs obviously increases the abundance of *Faecalibaculum* and *Desulfovibrio* and reduces the abundance of *Alistipes*, *Helicobacter* and *Rikenella*, which are associated with oxidative stress and inflammation.

**Table 2:** The changes in gut microbiota and microbial metabolites in neurodegenerative diseases.

Polyphenols	Diseases	Models	Composition of Gut Microbiota	Changes of Microbial Metabolites	Functions	Reference
Isoorientin	AD	APP/PS1 mice	in the fecal microbiota: dominated by the class *Mollicutes*, family *Prevotellaceae*, and genus *Prevotellaceae* UCG 001. in the cecal microbiota: dominated by the *phylum Proteobacteria* (*Pasteurellales*: *Pasteurellaceae*).	——	decreased Aβ plaque deposition in the cortex and hippocampus of AD mice; TNF-α ↓, IL-6 ↓, IL-4 ↑, IL-10 ↑; iNOS ↓, COX-2 ↓, ROS ↓	[42]
Quercetin	sporadic AD	streptozotocin-induced neuropathy rats	increased *Actinobacteria* at phylum and class level; increased the abundance of *p_Actinobacteria* and *c_Actinobacteria;* decreased the abundance of *f_Porphyromonadaceae*, *f_Oxalobacteraceae*, *g_Oxalobacter* and *g_Klebsiella*,	——	prevented myelin and axonal damage; ROS ↓	[43]
Quercetin-3-O-Glucuronide	AD	intracerebroventricular injection of Aβ1-42 induced AD mouse model	increased the abundance of *g_Barnesiella* and *g_Lactobacillus*; decreased the abundance of *g_Alistipes* and *g_Rikenella*	SCFAs ↑	alleviate spatial memory impairment; Aβ accumulation ↓, tau phosphorylation ↓;	[44]
Fisetin	PD	mouse model of PD induced by 1-methyl-4-phenyl-1,2,3,6-tetrahydropyridine (MPTP)	increased the abundance of *Lachnospiraceae*; decreased the abundance of uncultured_bacterium_g_Escherichia-Shigella and uncultured_bacterium_g_Bacillus	——	improve behavioral impairments, tyrosine hydroxylase ↑	[46]
Anthocyanins	NDD	High-fat diets induced neuroinflammatory in mice	increased the abundance of *Pseudoflavonifractor* and *Sporobacter*	Tryptophan and kynurenic acid ↑	attenuate neuroinflammation	[48]
	NDD	High-fat diets induced neuroinflammatory in mice	increased the abundance of *Bifidobacterium*, *Lactobacillus*, *Roseburia*, *Faecalibaculum*, *Parabacteroides* and *Ruminiclostridium*, and decreased the abundance of *Staphylococcus*	SCFAs: butyrate↑	SOD ↑, GSH-Px ↑; 5-HT ↑, dopamine ↑	[49]
Curcumin	AD	APP/PS1 mice	increased the abundance of *Bacteroidaceae*, *Prevotellaceae* and *Lactobacillaceae*, and decreased the abundance of *Rikenellaceae* at family level; increased the abundance of *Parabacteroides*, and decreased the abundance of *Prevotella* and *Bacteroides decreased* at genus level	——	improved the ability of learning; accumulation of Aβ ↓	[52,53]
Curcumin	PD	mouse model of PD induced by 1-methyl-4-phenyl-1,2,3,6-tetrahydropyridine (MPTP)	increased the abundance of *Muribaculaceae*, *Lactobacillaceae*, *Lachnospiraceae* and *Eggerthellaceae*, and decreased the abundance of *Aerococcaceae* and *Staphylococcaceae*	tyrosine, methionine, sarcosine and creatine ↑	improved motor deficits; glial cell activation ↓; the aggregation of a-synuclein (a-syn) ↓; dopa in the brain ↑	[55]
Resveratrol functional selenium nanoparticles (Res@SeNPs)	AD	mouse model of AD induced by aluminum chloride (AlCl_3_) and D-galactose(D-gal)	increased the abundance of *Faecalibaculum* and *Desulfovibrio*, and reduced the abundance of *Alistipes*, *Helicobacter* and *Rikenella*	——	improves cognitive dysfunction; Aβ aggregation ↓; ROS ↓, IL-10 ↑	[62]

Note: ↑: increased; ↓: decreased; ——: not mention.

### 3.2. Polyphenols Influence the Metabolites of Gut Microbiota in Neurodegenerative Diseases

Various metabolites can be produced by intestinal microbiota and then influence the function of the central nervous system. The neuromodulators include neurotransmitters, hormones, precursors of neurotransmitters and hormones, SCFAs, etc. Some metabolites enter the intestinal barrier into the circulation, cross the blood‒brain barrier and ultimately modulate nervous system function; these metabolites include SCFAs, tryptophan, tyrosine, gamma-aminobutyric acid (GABA) and brain-derived neurotrophic factor (BDNF) [63,64,65].

The gut microbiota can regulate the synthesis of several neurotransmitters and can directly produce neurotransmitters. For example, *Candida*, *Streptococcus*, *Escherichia* and *Enterococcus* species can produce 5-HT; *Bacillus* and *Serratia* species can produce dopamine; *Escherichia*, *Bacillus* and *Saccharomyces* species can produce noradrenaline; *Lactobacillus* species can produce acetylcholine; and *Lactobacillus* and *Bifidobacterium* can secrete GABA [66,67,68]. The gut microbiota can indirectly induce the production of neurotransmitters. Studies have shown that most 5-HT in the human body is produced by enterochromaffin cells in the gut. Recent studies have shown that SCFAs produced by intestinal microbiota are required to induce the production of colon 5-HT by enterochromaffin cells [69]. Although these gut neurotransmitters cannot cross the blood‒brain barrier, they may act on the vagus nerve or affect peripheral signals, ultimately affecting brain functions. Moreover, metabolites produced by intestinal microbiota can be used as precursors to synthesize the transmitters of the central nervous system. For example, tryptophan produced by the gut microbiota can penetrate the intestinal barrier and blood‒brain barrier before reaching the central nervous system to synthesize neurotransmitters. In addition, gut microbiota and specific bacteria have been shown to regulate several signaling pathways in the central and peripheral systems by regulating neurotransmitters and their receptors. For example, studies have shown that the levels of 5-HT, norepinephrine, dopamine and related receptors in the brain were significantly different in germ-free (GF) mice compared to normal mice. The gavage of normal mice with *Lactobacillus rhamnosus* JB-1 induced changes at the level of the GABA receptors in specific brain regions of mice [70].

Therefore, polyphenol intake plays an important role in neurodegenerative diseases by altering the gut microbiota and their metabolites.

#### 3.2.1. SCFAs

SCFAs, including acetate, propionate and butyrate, are a class of end products of bacterial fermentation of foods such as carbohydrates, proteins and fats. SCFAs have a critical role in the physiological function of the host brain. Studies have shown that SCFAs regulate the permeability of the blood–brain barrier (BBB). The BBB was more permeable in GF mice than in SPF mice due to the reduced expression of endothelial tight junction (TJ) proteins in the BBB. Colonizing *Clostridium tyrobutyricum* or *Bacteroides thetaiotaomicron* in mice can restore the integrity of the BBB by promoting the expression of TJ proteins. Further studies have shown that butyrate produced by the gut microbiota promotes the restoration of the integrity of the BBB. In addition, the abundance of the *Prevotellaceae* genus, which produces SCFAs, is significantly reduced in PD [71]. PD patients show reduced levels of acetic, butyric and propionic acid in fecal samples compared with age-matched controls [71,72]. In AD patients, decreased butyrate together with increased acetate and valerate were found to be closely related to the release of inflammatory factors, which may further damage the integrity of the tissue–blood barrier and ultimately allow inflammatory factors to enter the CNS and facilitate the pathological process of AD [73].

However, polyphenol intervention increases SCFA production by promoting the abundance of specific bacteria. Therefore, supplementation with polyphenols can resist the progression of NND by increasing the level of SCFAs. For example, quercetin-3-O-glucuronide intervention alleviated cognitive deficits in Aβ1-42-treated AD mice, significantly reversed the reduction in total SCFA levels in Aβ-treated mice, and markedly increased the levels of acetic and propionic acids [44]. Another study showed that anthocyanin intervention attenuated the high-fat diet-induced neuronal function decline in mice and, together with the increased abundances of SCFA-producing bacteria such as *Roseburia*, *Faecalibaculum* and *Parabacteroides*, especially *Roseburia*, which produces butyrate, was enhanced in anthocyanin-treated mice [49].

#### 3.2.2. Tryptophan

Tryptophan is an essential amino acid in mammals and is involved in various physiological processes, such as neuronal function, immunity and intestinal homeostasis. The three main metabolic pathways of tryptophan in vivo are the kynurenine metabolic pathway, 5-HT metabolic pathway and indole derivative metabolic pathway, among which the kynurenine metabolic pathway is the most important metabolic pathway. Approximately 90% of tryptophan is metabolized to kynurenine, and the rest is oxidized and decarboxylated to form 5-HT and metabolized by microorganisms to form indole derivatives [74,75]. However, kynurenine and its metabolic products show different biological effects, such as neuroactivity and neurotoxicity. Among them, the metabolite quinolinic acid (QUIN) is a competitive agonist of the N-methyl-D-aspartic acid (NMDA) receptor in the CNS. Excessive activation of NMDA by quinolinic acid can produce reactive oxygen species and damage and affect the function of neurons. Kynurenic acid (KA) has a neuroprotective effect against the neurotoxicity caused by quinolinic acid overexcitement at the NMDA receptor. In addition, kynurenic acid (KA) can also noncompetitively antagonize the α7-nicotinic acetylcholine receptor (α7-nAChR) and reduce extracellular glutamate (Glu) and dopamine levels. Several studies have shown that elevated tryptophan metabolism via the kynurenine pathway is associated with the pathophysiological processes of NND. Postmortem examination revealed that tryptophan 2,3-dioxygenase (TDO), quinolinic acid, amyloid-beta plaque and tau neurofibrillary tangle deposition were co-present in the hippocampus of AD patients [76]. The level of 3-hydroxy-kynurenine was significantly increased in the frontal cortex, putamen and substantia nigra compact areas, while the level of kynurenic acid (KA) was decreased in Parkinson’s disease patients [77]. However, anthocyanin intervention can modulate gut microbiota composition and promote tryptophan metabolism and increase the production of the neuroprotective metabolite kynurenic acid, which contributes to anti-neuroinflammation [47].

#### 3.2.3. Tyrosine

Tyrosine is a nonessential amino acid that can be produced from phenylalanine or obtained directly from the natural diet. Studies have shown that tyrosine and phenylalanine are precursors of catecholamine neurotransmitters such as dopamine, norepinephrine and epinephrine. Therefore, tyrosine plays an important role in the synthesis and utilization of neurotransmitters that take it as a precursor. Tyrosine affects the rapid activation of catecholaminergic neurons and has been shown to be useful in treating PD and depression [78]. It has been demonstrated that an elevation of tyrosine concentrations in the brain could rapidly stimulate and increase the synthesis and release of dopa and dopamine. For instance, Cui et al. reported that supplementation with curcumin increased the levels of tyrosine and dopamine in the brain. Further study showed that curcumin activated tyrosine-dopa metabolism and ultimately promoted dopamine increase, thus playing a protective role in PD [55]. Furthermore, supplementation with curcumin can increase tyrosine levels, which are closely related to the abundance of the *Lactobacillaceae*, *Aerococcaceae* and *Staphylococcaceae genera* [55].

## 4. Conclusions and Prospects

In recent years, many studies have elucidated the potential role of plant-derived polyphenols in the treatment of NND, including AD and PD. Some pathophysiological impairments in neurodegenerative diseases can be ameliorated by the dietary intake of polyphenols. Studies have shown that polyphenols can significantly affect the intestinal microbiome and regulate its bacterial structure and function. Ingestion of polyphenols is beneficial to the development of the gut microbiome and the health of the host. In addition, the metabolites of polyphenols by biotransformation of intestinal flora may regulate the functions of the central nervous system (Figure 1). At present, the properties of intestinal microflora in patients with neurological diseases are not fully understood, and the mechanism of interaction between microflora and host is still the focus of research. Although our current understanding of the specific mechanisms is limited, dietary intervention and the regulatory functions of microbes are promising potential therapeutics for several neurological diseases. A thorough understanding of the mechanism and pathways of polyphenol absorption and metabolism in the body may become a potential effective strategy to treat and prevent neurological diseases by microorganisms. Therefore, there is an urgent need for more precise microbiome sequencing in this area of research to determine the exact influence of the gut microbiome on polyphenol metabolism and its beneficial effects on health. Moreover, the use of more precise metabolomic analysis provides a powerful new tool for studying the beneficial effects of plant polyphenols and the specific metabolic mechanisms mediated by the gut microbiome, as well as the overall positive effects on the host.

## Figures and Tables

**Table 1:** Biotransformation of polyphenols by gut microbiota.

Polyphenols	Composition of Gut Microbiota	The Metabolites of Polyphenols	Reference
Curcumin	firmicute *Blautia* sp. *(MRG-PMF1)*, *Escherichia fergusonii* (ATCC 35469), and two *E. coli strains* (ATCC 8739 and DH10B)	Demethylcurcumin, bisdemethylcurcumin, dihydrocurcumin, tetrahydrocurcumin, and ferulic acid	[20]
Quercetin and rutin	*Eubacterium ramulus*, *Clostridium orbiscindens*, *Eubacterium oxidoreducens*, *Butyrovibrio* spp., *Bacteroides fragilis*, *Eubacterium ramulus*, *Clostridium perfringens*, *Bacteroides JY-6*, *Bifidobacterium B-9*, *Lactobacillus L-2*, and *Streptococcus S-2*	homo-procatechuic, protocatechuic, 4-hydroxybenzoic, and 3-(3-hydroxyphenyl) propionic acids	[23,24,25]
Daidzein and genistein	*Lactobacillum*, *Bifidobacterium* and *Bacteroides;* *Lactococcus strains, E. faecium* INIA P455 and *L. paracasei* INIA P461; *Eggerthella* sp. YY7918, *Eubacterium ramulus* and *Clostridium* sp. HGH 136	S-equol, 2-(4-hydroxyphenyl)-propionic acid, and O-desmethylangolensin (O-DMA)	[8,27,28]
Resveratrol	*Bifidobacteria infantis* and *Lactobacillus acidophilus*; *Slackia equolifaciens* and *Adlercreutzia equolifaciens*	dihydroresveratrol	[30]
Anthocyanins	*Lactobacilli* and *Bifidobacteria* increased; *Staphylococcus aureus* and *Salmonella typhimurium* reduced; *Eubacterium ramulus* and *Clostridium saccbarogumia*	protocatechuic acid, gallic, syringic, vanillic, and p-coumaric acids	[33]
Ellagitannins	*Gordonibacter* genus and *Clostridium coccoides* group	urolithins (Uros)	[36]
Proanthocyanidins	*Adlercreutzia equolifaciens JCM 14793T*, *Eubacterium* sp. *SDG-2*, *Eggerthella lenta rK3*, *Eggerthella lenta CAT-1*	(−)-epigallocatechin (EGC), (−)-gallocatechin (GC), (±)-epicatechin (EC), and (±)-catechin (C)	[39]

## Data Availability

Not applicable.
